# The construction of the health professional in palliative care contexts: a scoping review on caring for the person at the end of life

**DOI:** 10.1016/j.pbj.0000000000000010

**Published:** 2018-07-03

**Authors:** Vitor Parola, Adriana Coelho, Álvaro A. Romero, Roland P. Peiró, Joan Blanco-Blanco, João Apóstolo, Montserrat Gea-Sánchez

**Affiliations:** a Abel Salazar Biomedical Sciences Institute, University of Porto, Porto; b Nursing School of Coimbra, Health Sciences Research Unit: Nursing, Portugal Centre for Evidence-Based Practice: A JBI Centre of Excellence, Coimbra, Portugal; c Faculty of Nursing and Physiotherapy, GESEC, Lleida University, Lleida, Spain.

**Keywords:** end of life care, health care worker, literature review, palliative care, staff attitude

## Abstract

**Aim:**

The aim of the study was to map of the literature on the elements contributing to the construction of the health care professional in the context of palliative care.

**Methods:**

Scoping review based on Arksey and O’Malley framework. PubMed, Embase, CINAHL, Scopus databases, and gray literature were the sources searched (2005–2015), completed by reference searching, hand searching, and expert consultations. Primary studies focusing on different professionals working in palliative care units or hospice centers were eligible for inclusion.

**Results:**

From a total of 3632 articles, 22 met the inclusion criteria. The content of the studies was described and classified in 5 elements: (i) construction and application of the concept of care; (ii) psychosocial effects that the daily care produces; (iii) working conditions that influence the caregiving provided; (iv) knowledge mobilized in the provision of care; and (v) strategies adopted by health care professionals to build relationships. Data about nurses, physicians, and psychologists were found, but no data were found about social workers. Gaps identified in the publications were as follows: relationship competencies and strategies adopted; the real needs from educational programs; and the view of other professionals.

**Conclusions:**

Key elements identified in the concept of the construction of the health care professional should be addressed in future interventions: prevention of emotional exhaustion, depersonalization, and achievement of a greater personal accomplishment. In addition, none of the articles retrieved offered the different perspectives of all the disciplines in a multidisciplinary team.

## Introduction

As a result of medical advances, life expectancy is progressively increasing. Consequently, the number of people living with a chronic disease has increased. This has contributed to a growing need for palliative care (PC), and therefore, more health care professionals (HCPs) will provide care at the end of life (EoL).^1^ The World Health Organization defines PC as “an approach that improves the quality of life of patients and their families facing the problems associated with a life-threatening illness, through the prevention and relief of suffering by means of early identification and impeccable assessment and treatment of pain and other problems, physical, psychosocial and spiritual.”^2^

However, along with the perception that science has all the answers, doubts and restlessness arise on how to implement some of the knowledge when we come across patients at the EoL. Literature has shown that PC has been proven to be more effective than standard approaches, improving the quality of life of patients and their families at a lower cost.^3–7^ Nevertheless, contact with people at EoL is conducive to feeling intense emotions that evidence the fragility and limitations of human life. The important role of emotions in human life becomes even more important as death approaches.^8^

Hospital experiences that produce the greatest emotional impact are related to death, suffering, and caring for patients at the EoL.^9^ Taking into account that the above statement is significant because most people in developed countries die in hospitals; therefore, attention to the quality of life in these contexts has increased.^10–13^

Previous studies conclude that the HCPs who feel more competent and face the EoL with more personal resources provide more effective care^14,15^ and those who have more knowledge in PC score higher with regard to control of negative emotions and fear of death.^16^

Previous personal experience related to death can provide the HCPs with a better perception of the EoL of their patients and with better personal resources.^17^ In addition, HCPs who spend a greater percentage of time in contact with patients in EoL situation reported more positive attitudes toward death.^18^

It is known that effective communication is an element that allows the delivery of excellent health care, being an essential factor to face EoL care. In fact, patients at EoL valued HCP trained in communication skills.^19^ HCPs play a key role supporting the informal caregivers, providing them with knowledge and evaluating their needs.^20,21^

Although previous studies provide a representation of what happens at the EoL, they do not explain the elements involved in the construction of the HCP that provide EoL care. Moreover, if we want to achieve the main goals of PC,^2^ we must have highly qualified HCPs. It is therefore important to understand what factors are involved in the construction of this caregiver.

As the population continues to age and PC becomes more present in health care practice, having an understanding of the necessary elements of HCPs, who effectively manage care in EoL, is essential.

HCPs in PC are working daily with people who are dying and are exposed to intense emotional reactions. Therefore, it is essential to understand the elements that contribute to the construction of the HCP in this unique context.

To date, despite the unique nature of this context, no review has examined the specific elements that contribute to the construction of the HCP in the context of the PC, which supports the need for this scoping review.

### Aims

The aim of this scoping review is to examine the extent, range, and nature of the research activity around which elements contribute to the construction of the HCP in the context of the PC and to identify research gaps in the existing literature.^22^

## Methods

This scoping review was guided by the methodology proposed by the Joanna Briggs Institute Scoping Reviews, and is based on the framework by Arksey and O’Malley^23^ for conducting scoping reviews. It takes into account the works by Levac et al^24^ and Daudt et al,^22^ which included: (i) identifying the research question; (ii) finding relevant studies; (iii) selecting appropriate studies; (iv) charting the data; and (v) collating, summarizing, and reporting the results.

### Inclusion criteria

This scoping review considered quantitative and qualitative primary studies published in English, Spanish, Catalan, and Portuguese during the last 10 years that focus on HCPs (physicians, nurses, social workers, and psychologists) working in palliative care units (PCUs) or hospice centers and caring for inpatients >18 years at the EoL.

### Search strategy

According to different terms and rules for searching in each database, the effective combination of search terms was designed by one reviewer (RPP) (librarian expert in health science) and discussed with 3 other reviewers (VOP, AAR, and JBB).

Once relevant material was selected from electronic literature databases (CINAHL Plus, PubMed, Embase, Scopus, DART-Europe, OpenGrey, Grey Literature Report), relevant websites were searched, key journals were hand-searched, and reference lists were retrieved from articles, to increase our capture of relevant material. Finally, recommendations from experts in the field were also used to identify further published, unpublished, and ongoing studies. The process was documented in detail to enable the study to be replicated by others (Table 1).

**Table 1:**
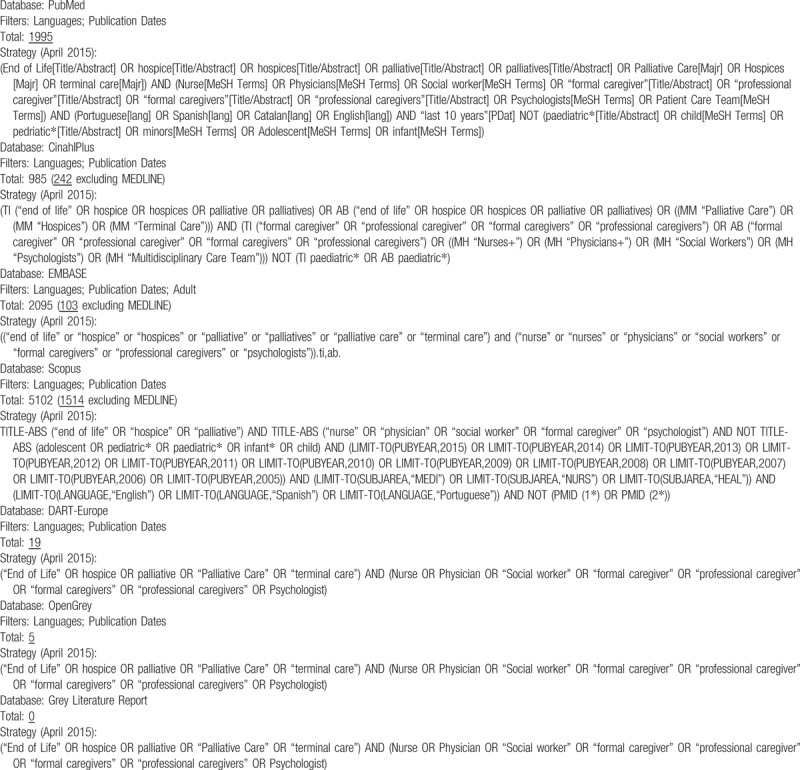
Final database search strategy

The Mendeley software was used to manage the list of all the articles retrieved and any duplication was removed.

### Selection criteria

The review pursues to identify all the published research about which elements contribute to the construction of the HCP in the context of the PC. Articles searched were assessed for relevance by 2 independent reviewers (VOP and ANC). Those that meet the inclusion criteria, based on the information provided in the title and abstract, were included. When the relevance of a study was unclear in the abstract, the full article was reviewed.

The full article was retrieved for all studies that met the inclusion criteria. Based on full texts, 2 reviewers (VOP and ANC) examined independently whether the studies met the inclusion criteria. The disagreements that arose between the reviewers were resolved through discussion, or with a third reviewer (MGS).

### Data extraction

Quantitative and qualitative data were extracted from papers in the review using a data extraction table, taking into account the review question (Table 2    ). In this process, 2 reviewers (VOP and ANC), independently of one another, charted the “first five to ten studies using the data-charting form and met to determine whether their approach to data extraction was consistent with the research question and purpose,” as suggested by Levac et al.^24^ Any disagreement was resolved through discussion, or with a third reviewer (MGS). In addition, when it was necessary, primary authors were contacted for further information/clarification of data.

**Table 2:**
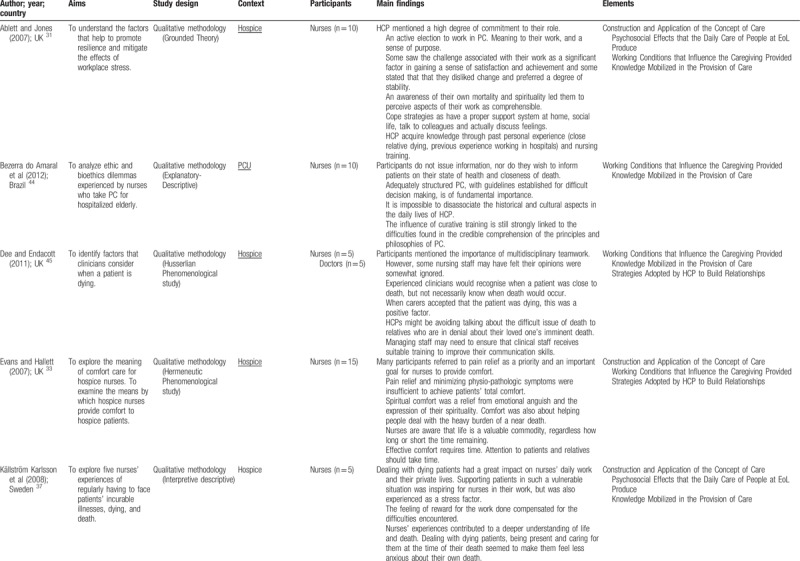
Extraction instrument

**Table 2 (Continued):**
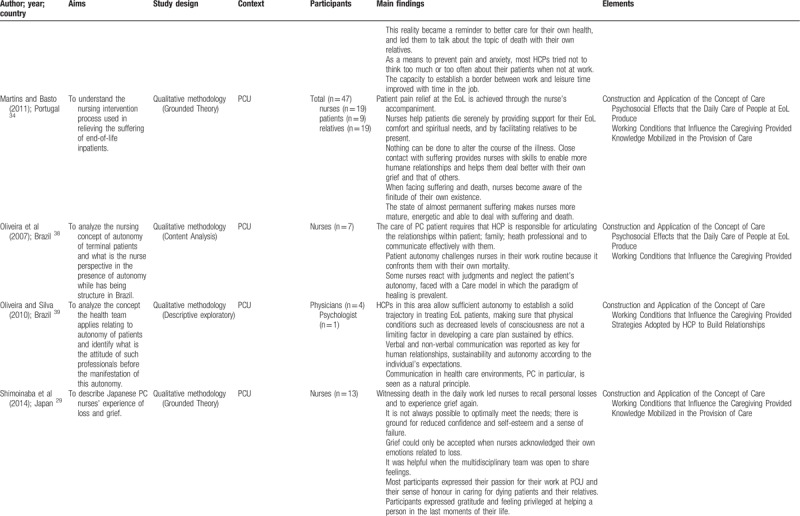
Extraction instrument

**Table 2 (Continued):**
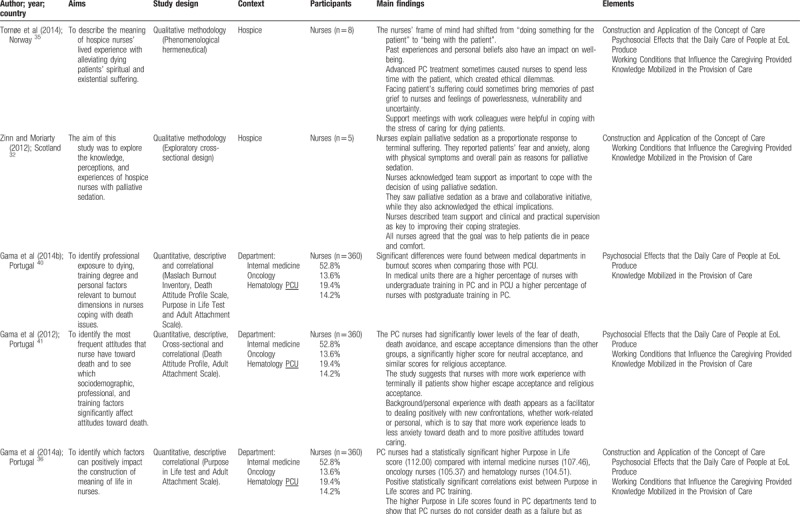
Extraction instrument

**Table 2 (Continued):**
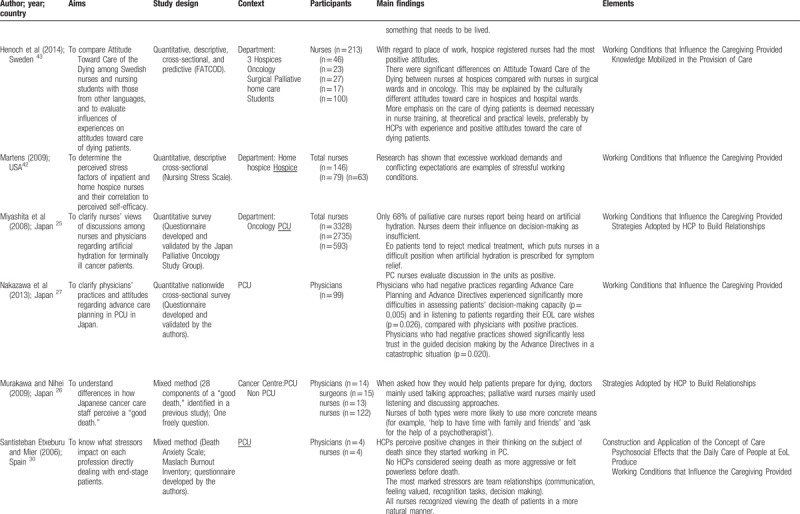
Extraction instrument

**Table 2 (Continued):**
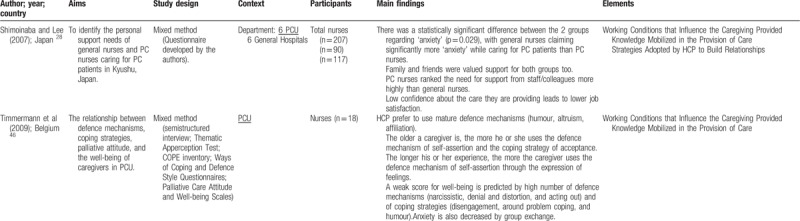
Extraction instrument

## Results

After the duplicates were removed, 3632 records were identified for study selection. A total of 163 documents met the inclusion criteria, based on the titles and abstracts; therefore, the full-text articles were obtained. Full-text articles were read, after which 22 fulfilled the inclusion criteria. As a result, 22 studies were analyzed.

**Figure 1:**
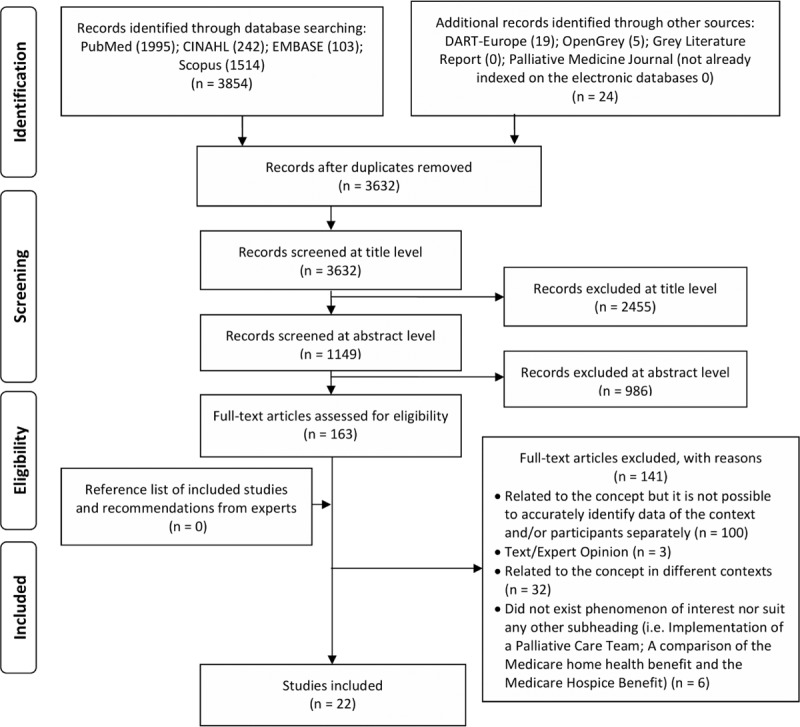
PRISMA flow diagram of the scoping review process.

The stages of the scoping review process can be seen in the PRISMA flow diagram (Fig. 1).

The overview of the reviewed material is presented and discussed in narrative form. Tables and figures are included to aid in data presentation.^23^ The overview of the studies included is presented in Table 2    .

About the geographical source 9 studies were conducted outside Europe (n = 9): the United States, Brazil, and Japan.^25–29^ The other 14 were from the United Kingdom, Spain, Belgium, Norway, Scotland, Sweden, and Portugal.

Regarding the year of publication, most of the articles had been published between 2007 and 2014 (n = 21), 1 had been published in 2006,^30^ and none had been published in 2005 or 2015 (until April). Regarding the research methods used, the majority of the studies were qualitative (n = 12), 4 collected quantitative data, and another 4 studies used mixed methods.

Data about nurses were found in twenty out of the twenty two articles included, four articles had data about physicians, one had data about psychologists, and no data were found about social workers. From the 22 articles identified, 14 were conducted in PCUs and the other 8 in inpatients hospices.

The content of the studies was described and classified in 5 main elements identified in the articles: (i) construction and application of the concept of care (including what caring for HCPs means, how they provided this care, and with what objectives. It also includes the factors that intervened in the development [construction] of this meaning, provision, and purpose); (ii) psychosocial effects that daily care produces (including the personal, professional, emotional, and psychological repercussions that come from working in PC); (iii) working conditions that influence the caregiving provided (including facilitating factors and difficulties that are extrinsic to the professional, but that influence the care provided, such as the time available for caring, teamwork, or the paradigm of healing still prevalent in some professional teams); (iv) knowledge mobilized in the provision of care (including not only academic knowledge but also the knowledge that derived from the professional experience and the personal experience of each professional; and (v) strategies adopted by HCP to build relationships (including the strategies adopted to build relationships with the team and the patient). All these elements are interrelated and mutually influential, and none of these elements is absolutely distinct from others, existing the possibility of a cross between them.

### Construction and application of the concept of care by HCPs

HCPs mention that for them care means a sense of purpose and honor and devote a high degree of commitment to their role.^29,31^ The findings of the studies included mention that the goal of care is to provide a peaceful death. They also mention total pain as a reason for PC sedation and that the care provided should ensure that the bereaved would not have memories of their loved ones dying in distress.^32^

Therefore, to achieve freedom from pain is one of the priorities of comfort care, but pain relief and minimizing the pathophysiological symptoms were insufficient for the patients’ fully experience of comfort.^33^ Care means to provide calm and relaxation, sometimes with a silent compassion and caring touch, a feeling of rest and freedom from the mental and emotional distress. It is about alleviation of patient suffering, relief from mental and emotional distress, and the different ways in which people express their spirituality and their fear of death and dying. It means providing care in a physical, emotional, and spiritual way, not only helping patients to live but also helping patients to die serenely. The mental focus of care has shifted from doing something for the patient to being with the patient and enhancing their quality of life. The focus is now on the individual, and the concept of care has a holistic approach.^31,33–35^

HCPs report not considering death as a failure but as something that needs to be lived; this perspective gives them a motivation to find the meaning of care in their work. This sense of commitment also influences caregiving.^36^ HCPs were still concerned about life as the most valuable commodity, regardless of the time remaining.^33^ Furthermore, considering death as a part of life became an expression of their consideration for life itself as a cycle.^29^ HCPs’ experiences of care contributed to greater understanding of death as a part of life^37^; death was not seen as something aggressive, and they did not feel powerlessness before death.^30^ The explanation could be the experience that nothing could be done to change the outcome of the disease.^34^

Such reality shows that it is possible to care for patients at the EoL differently and make death more human while fostering one's personal development.^36^ Comforting the patients in their vulnerable EoL situation, recognizing patient autonomy, and providing a care sustained by ethics were the motivation that guided their work; they feel passion for their work in this context even when it is seen as stressful.^29,37–39^

### Psychosocial effects that the daily care of people at EoL produces in HCPs

The daily care of EoL patients spurs an awareness of the HCPs’ own spirituality, mortality, and finitude of their own existence.^31,34,37,38^ Findings show that this perception led to reflect about the meaning of life and death. Accompanying the patients at the moment of death brought on less anxiety about their own death. Participants expressed that this experience makes them more aware and understanding, and leads them to bringing up the subject of death when talking with their own families.^35^ Through this experience, HCPs gain greater human and communication competency. Moreover, their close witnessing of death becomes a reminder of the importance and meaning of taking care of their health.^34,37^ They perceive positive changes in their thinking on the subject of death since their work in PC.^30^ We need also to be aware that facing patients’ suffering could sometimes trigger feelings of uncertainty and vulnerability.^35^

Gama et al^40^ show that PC nurses had a statistically significant higher Purpose in Life score (112.00) compared with internal medicine nurses (PIL 107.46, *t* = 2.07, *P* < .040), oncology nurses (PIL 105.37, *t* = 2.52, *P* < 0.013), and hematology nurses (PIL 104.51, *t* = 2.98, *P* < 0.003), and also lower burnout scores when comparing those medical departments with PCUs (significant lesser levels of emotional exhaustion [*t* = 2.71; *P* < 0.008], depersonalization [*t* = 3.07; *P* < 0.003], and higher levels of personal accomplishment [*t* = 2.24; *P* < 0.027]).^36^ This is in accordance with the article which mentions that palliative HCP had significantly lower levels in the fear of death, death avoidance, and escape acceptance dimensions than the other departments, a significantly higher score for neutral acceptance, and similar scores for religious acceptance.^41^

### Working conditions that influence the caregiving provided

Excessive workload demands and conflicting expectations are examples of stressful working conditions for nurses.^25,42^ Studies mentioned that past personal experience, professional experience (more work experience leads to less anxiety toward death and to more positive attitudes toward caring), and the establishment of personal and professional boundaries help to deal with these issues when providing care.^31,35,41,43^ HCPs become skilful in coping with the suffering of others and their own,^34^ and gain awareness of how meaningful the relationships established through their role can become.^29^

Nonetheless, it is impossible to disassociate the historical and cultural aspects in the daily lives of HCPs, and it is a constant challenge due to their own prejudices and values.^38,43,44^ The influence of curative training is still strongly related with the difficulties found for the real understanding of the principles and philosophies of PC.^36,44^ In a health care model in which the paradigm of healing is prevalent, some professionals react with judgments and neglect the patient's autonomy.^38^

The importance of multidisciplinary teamwork was mentioned too. However, 2 of the articles suggested that some HCPs may feel that their opinions are not considered, which could be frustrating and affect the caregiving.^29,45^ One study even mentioned that team relationships are the most marked stressors,^30^ instead of being an opportunity for sharing experiences and looking for support.^36^

In addition, communication skills were mentioned as a factor that could improve the care provided. Communication, particularly in PC, is viewed as a natural principle, and communication skills training should be provided by the managers.^32,35,39,45^

Other fundamental factors that influence the caregiving in PC are as follows: an adequately structured PC, with guidelines established for difficult decision making^44^; the participation in the decision-making process, and how the professional is closely involved^25^; and time because comfort care takes time. Time is needed to develop a sense of dependability with patients and families, to establish meaningful communication, and to deliver sensitive and effective clinical care. Taking the time to be present, ensuring that people are made to feel that they “matter” when they reach the point of greatest overcoming vulnerability.^33,35,36^

Physicians, who displayed negative practices regarding advance care planning and advance directives, found significant added difficulties in appraising the patient's decision-making capacity (*P* = .005), letting the patients express their wishes for EoL care (*P* = .026) and lower confidence in treatment decisions in a catastrophic situation (*P* = .020), compared with physicians with positive practices.^27^

One of the studies mentioned that low confidence in the care provided leads to lower job satisfaction.^28^ As it is not always possible to address a numerous variety of needs to fully satisfy patients, trust is likely to be reduced, as self-esteem, and sometimes one may experience a feeling of failure as a palliative professional. This could cause doubts as to whether or not the full professional ability has been displayed to support patients, and could influence the way care is provided.^29^ Confidence, trust, and experience are necessary in the provision of care,^32^ meaning that it seems essential that HCPs use defense mechanisms and coping strategies simultaneously.^32,46^

The last factor mentioned by the included studies was the support from staff, including the unit manager, educator (supervisor) colleagues,^28,32,35^ family and friends.^28^

### Knowledge mobilized in the provision of care

In the provision of care, HCPs need to possess several coping strategies such as having a good support system at home, social life, psychological strength, balance of mind and body, and assuming responsibility in caring for their own feelings by talking to colleagues and discussing their feelings or how to manage cases.^28,31^ Furthermore, as previously mentioned, the HCP knowledge and personal experiences have an important influence in the care provided,^29,32,43,44^ for example, being able to use silence for therapeutic and comforting purposes.^35^

In fact, HCPs with more work experience with terminally ill patients had higher escape and religious acceptance, which is explained by the developed strategies in dealing with their emotional response to death and dying.^41^

For example, after having worked for an extended period, they improved the ability to clearly differentiate between work and leisure time.^37^ An experienced clinician would acknowledge when a patient's EoL is near and take the necessary action to accompany a patient during the last hours of their life.^29,34,45^ Personal experience with death appears as a facilitator to dealing positively with new confrontations, whether work-related or personal.^41,46^

A study shows that there is a higher percentage of nurses with undergraduate training in PC in medical units such as hematology; internal medicine; and oncology, than in PCUs, where there is a higher percentage of nurses with postgraduate training. Further training seems to provide more coping strategies in dealing with EoL care, as PC professionals had less levels of burnout compared with other departments.^40^ As a positive statistically significant correlation between Purpose in Life scores and PC training was also found, knowledge about coping strategies seems to be important.^36^

### Strategies adopted by HCPs to build relationships

Dee and Endacott^45^ and Shimoinaba and Lee^28^ mentioned that seeking the support of colleagues could be a way to develop a strong relationship between team members. One way to build relationships was to help people “to deal with the heavy burden of death awareness by entering into their world in a compassionate and connected interpersonal relationship.”^33^ HCPs also recommend more active communication between team members.^25^ Verbal and nonverbal communication was considered key in mediating between relatives, individuals, and professionals.^39^ Palliative HCPs mainly used listening and discussing approaches to help patients prepare for death. They were more likely to use more concrete means than nonpalliative professionals.^26^

## Discussion

This scoping review identifies 5 key elements around the construction of the HCP concept in the context of PC: construction and application of the concept of care; psychosocial effects that daily care produces; working conditions that influence the caregiving provided; knowledge mobilized in the provision of care; and strategies adopted by HCP to build relationships; and it also identifies research gaps in the existing literature such as the absence of the perspective of all the members of the multidisciplinary team.

As mentioned by HCPs in the studies included care means a sense of purpose and honor with a high degree of commitment to their role.^29,31^ Nevertheless, other studies showed that the experience of witnessing patients’ dying and death overtime had a great impact on the professionals’ day-to-day work and in their private lives, and is also seen as stressful. In a way of preventing grief and anxiety, most professionals “tried to avoid thinking too much, too often, or too intensely about their patients when they were not at work.”^37^ However, it seems that the feeling of reward and recognition obtained through their commitment compensated for any difficulties and was even the inspiration that guided their work.^37^

From the analysis of the included articles, it seems that the daily care of people at EoL is not affecting the HCP at a high level compared with other units. The levels of burnout are lower in PC professionals,^40^ as they undergo positive changes in their perception of the subject of death from their work in a PC context. However, it is necessary to interpret the data (self-reported questionnaires), through the participants’ contribution.

Miyashita et al^25^ mention that sometimes PC nurses are put in uncomfortable situations by physicians. However, data were gathered retrospectively on nurses’ views only, which might be subject to incomplete or mistaken recalling and does not take into consideration the view of other professionals. Further studies should address which strategies should be adopted to deal with such an uncomfortable situation. In this sense, it is important to mention that the sample of most included studies is constituted by nurses, which is perhaps justified by nurses constitute the HCP that remains more time with patients, ensuring its caring 24 hours a day.^47–49^

HCPs might be avoiding talking about the difficult issue of death to relatives who are in denial about their loved one's imminent death, and therefore appropriate communication skills training is required.^45^ Even mentioning the importance of these programs, it is still not clear which are the real needs or strategies that need to be adopted. As mentioned in Henoch et al,^43^ a first step could be to take into consideration that both theoretical and practical education should be provided, preferably under the supervision of HCPs with positive attitudes toward care of dying patients.

HCPs seem to acquire greater human and communication capacities^34^; however, the literature did not mention precisely those relationship competencies. HCPs are responsible for articulating the relationships between patient, family, and other HCPs, and to communicate effectively with them, but the reality is that the strategies they use in this process are not clear.^38^

The most marked stress factors, however, seem to be team relationships. It seemed that some nursing staff were feeling somewhat ignored in their opinions, which could be a source of frustration.^30,45^ Further investigations should try to provide understanding on which strategies should be adopted to avoid this element of stress.

The element of communication is considered as a natural element of team cohesion and a means of influencing others.^39^ HCPs should enter into their patients’ worlds in a kind-hearted way and have an interpersonal relationship; they therefore need time for meaningful communication.^33^

Another aspect to be discussed is the fact that this scoping includes studies conducted in 10 countries; however, the countries with the most articles included are Japan (5 studies) and Portugal (4 studies). This data is curious given that Japan was one of the first countries which emerged PCUs (70s)^50^ and is one of the countries that has PC well integrated into his health care system.^51^ Portugal, for its part, where CP started only in the 90s,^50^ is recognized for actively developing PC in the country.^51^ Perhaps this data (although distinct) justifies the high research activity in these 2 countries. Although in all the included studies the elements can be considered universals, in these 2 countries it is noteworthy that none of the studies published in Japan addresses the element “Psychosocial Effects that the Daily Care of People at EoL Produce,” and none of the studies published in Portugal addresses the element “Strategies Adopted by HCP to Build Relationships,” which is possibly due to cultural issues.

This study is unique in including publications not only in English but also in Spanish, Catalan, and Portuguese, thus broadening the scope and enabling these cultural comparisons.

### Limitations of the scoping review

This scoping review has some particularities that limited our capacity to comprehensively understand the problem under analysis.

In the articles mentioning social workers these had been included in larger samples with participants who did not fit our inclusion criteria and had not followed the analysis separately.^30^ Most of the studies analyzed in this scoping review were not carried out taking into account all team members of the multidisciplinary team. In 18 articles data were only gathered retrospectively on nurses’ views. Although we use the term HCPs because our study is centered upon the multidisciplinary team, most of the data relate to nurses. Therefore, this limits any possible generalization among other HCPS involved in the EOL care.

It was not the main purpose of this scoping review to examine which palliative team members were the focus of the studies; however, our results suggest that it would be positive to incorporate other team members’ perspectives into PC team discussions to inform about their work and to have an input in drawing up multidisciplinary team planning.

In addition, the scoping review included studies with imbalanced sample sizes and concerns exist about the rigor of study designs. However, a clear research question, a clear research strategy, and appropriate and strong inclusion criteria provide validity to its findings.^52^

Finally, due to the fact that the number of included studies is high, and a broader description would have repercussions on a table of high dimensions, the findings presented in Table 2     are only the main findings; however, in “Results” section we presented a more detailed exploration in narrative form.

### Implications for research

The analysis of the existing evidence does identify gaps in the literature, resulting in recommendations for future research.

It seems necessary in future research to understand whether the impact of care at EoL and the stress and anxiety suffered by HCP are ever compensated by the satisfaction, sense of commitment, and purpose of the work they do, compared with other health care departments. It is also important to understand whether the lower levels of burnout reported by PC professionals are specific to Portugal or they could be generalized to other countries. A systematic review of the literature focused on the prevalence of burnout could help in this understanding.

The state of the science on HCPs’ concerns and needs is evolving. Research evidence requires further research in these areas to find the best strategies and theories on how to provide this care in the most possibly efficient manner. An intentional focus on understanding the elements that contribute to the construction of the HCP in the specific context of PC is needed to improve not only the welfare of the HCP but also the care provided to the patient at EoL. This assumes a role of significant relevance for the caregiver and the patient who receives care at this singular moment.

It is also recommended the realization of studies that address the element “psychosocial effects that daily care produces” in the Japanese context, and studies that address the “strategies adopted by HCP to build relationships” element in the Portuguese context. This could be an important theme of analysis in future research because this lack of evidence incurs caregiving costs that may not be tangible. The empirical knowledge that facilitates the professional growth of PC teams should become a priority.

Finally, the literature reveals that working in PC leads to feelings of intense emotions, evidencing the fragility and limitations of human life, which could lead to several stressful and demanding challenges. However, comparing to nurses in other contexts, nurses working in PC have lower levels of burnout.^53^ This ambiguity demonstrates the importance to develop more primary studies to understand how nurses experience the caring in PC.

## Conclusions

This scoping review examined the extent, range, and nature of the research activity around which elements contribute to the construction of the HCP in the context of the PC and identifies 5 key elements: construction and application of the concept of care; psychosocial effects that daily care produces; working conditions that influence the caregiving provided; knowledge mobilized in the provision of care; strategies adopted by HCP to build relationships.

This scoping review contributes to the generation of a solid body of empirical knowledge that facilitates the professional growth of PC teams becausre it identifies key elements in the concept of the HCP construction, revealing the importance of developing specialized training programs and adding new elements to define strategies of action, showing the necessity of promoting interpersonal skills and emotional management mechanisms. It also reinforces the need to incorporate all team members’ perspectives, such as social workers, in PC team discussions, evidencing that none of the articles retrieved offered the perspective of all the disciplines of the multidisciplinary team.

## Acknowledgments

None.

## Author contributions

VOP participated in the design of the study, carried out the study and had the main responsibility for writing the manuscript. ANC, AAR, and MGS participated in conceiving the study and writing the manuscript. RPP, JAA, and JBB supported data analysis and writing the manuscript. All authors helped in revising and making substantial contributions to the manuscript, and also read and approved the final manuscript.

## Conflicts of interest

This research received no specific grant from any funding agency in the public, commercial, or not-for-profit sectors.

The authors declare no conflicts of interest.

## References

[1] Vos T Flaxman AD Naghavi M Years lived with disability (YLDs) for 1160 sequelae of 289 diseases and injuries 1990-2010: a systematic analysis for the Global Burden of Disease Study 2010. *Lancet* 2012; 380:2163–2196. 23245607 10.1016/S0140-6736(12)61729-2 PMC6350784

[2] World Health Organization National Cancer Control Programmes: Policies & Managerial Guidelines. 2nd ed Geneva:World Health Organization; 2002.

[3] Greer JA Jackson VA Meier DE Early integration of palliative care services with standard oncology care for patients with advanced cancer. *CA Cancer J Clin* 2013; 63:349–363. 23856954 10.3322/caac.21192

[4] Taylor DH Bull J Zhong X The effect of palliative care on patient functioning. *J Palliat Med* 2013; 16:1227–1231. 24020918 10.1089/jpm.2013.0040 PMC3791032

[5] von Gunten CF Evolution and effectiveness of palliative care. *Am J Geriatr Psychiatry* 2012; 20:291–297. 22367161 10.1097/JGP.0b013e3182436219

[6] Gomes B Calanzani N Curiale V Effectiveness and cost-effectiveness of home palliative care services for adults with advanced illness and their caregivers. *Cochrane Database Syst Rev* 2013; 6:280. 10.1002/14651858.CD007760.pub2 PMC4473359 23744578

[7] Hales S Zimmermann C Rodin G Review: the quality of dying and death: a systematic review of measures. *Palliat Med* 2010; 24:127–144. 20085963 10.1177/0269216309351783

[8] Peterson J Johnson MA Halvorsen B What is it so stressful about caring for a dying patient? A qualitative study of nurses’ experiences. *Int J Palliat Nurs* 2010; 16:181–187. 20559180 10.12968/ijpn.2010.16.4.47784

[9] Benbunan-Bentata B Cruz-Quintana F Roa-Venegas JM Nursing students’ coping with pain and death: a proposal for ameliorative action. *Int J Clin Heal Psychol* 2007; 7:197–205.

[10] Oliva MP Morir en España: El reto de una muerte digna;Atrapados en la tecnología;La muerte clandestina. *El País* 2005 1–17.

[11] Cohen J Bilsen J Addington-Hall J Population-based study of dying in hospital in six European countries. *Palliat Med* 2008; 22:702–710. 18715968 10.1177/0269216308092285

[12] Cohen J Houttekier D Onwuteaka-Philipsen B Which patients with cancer die at home? A study of six European countries using death certificate data. *J Clin Oncol* 2010; 28:2267–2273. 20351336 10.1200/JCO.2009.23.2850

[13] World Health Organization. The Solid Facts: Palliative Care. (Davies E, Higginson I, eds.). World Health Organization; 2004.

[14] Claxton-Oldfield S Crain M Claxton-Oldfield J Death anxiety and death competency: the impact of a palliative care volunteer training program. *Am J Hosp Palliat Care* 2007; 23:464–468. 10.1177/1049909106294882 17211000

[15] Cevik B Kav S Attitudes and experiences of nurses toward death and caring for dying patients in Turkey. *Cancer Nurs* 2013; 36:E58–E65. 23151504 10.1097/NCC.0b013e318276924c

[16] Trujillo-De Los Santos Z Paz-Rodríguez F Sánchez-Guzmán MA Estudio exploratorio sobre conocimientos de cuidados paliativos y actitudes de profesionales de la salud, ante la muerte y el trabajo emocional. *Rev Mex Neurocienc* 2013; 14:8–13.

[17] Black HK Rubinstein RL Direct care workers’ response to dying and death in the nursing home: a case study. *J Gerontol B Psychol Sci Soc Sci* 2005; 60 1:S3–S10. 15643044 10.1093/geronb/60.1.s3

[18] Dunn KS Otten C Stephens E Nursing experience and the care of dying patients. *Oncol Nurs Forum* 2005; 32:97–104. 15660148 10.1188/05.ONF.97-104

[19] Puente CP Furlong LV Gutiérrez JLG Estrategias de afrontamiento y personalidad resistente en pacientes de cuidados paliativos. Un estudio preliminar. *Clínica y Salud* 2005; 16:65–89.

[20] Harding R List S Epiphaniou E How can informal caregivers in cancer and palliative care be supported? An updated systematic literature review of interventions and their effectiveness. *Palliat Med* 2012; 26:7–22. 21737481 10.1177/0269216311409613

[21] Docherty A Owens A Asadi-Lari M Knowledge and information needs of informal caregivers in palliative care: a qualitative systematic review. *Palliat Med* 2008 153–171. 18372380 10.1177/0269216307085343

[22] Daudt HML van Mossel C Scott SJ Enhancing the scoping study methodology: a large, inter-professional team's experience with Arksey and O’Malley's framework. *BMC Med Res Methodol* 2013; 13:48. 23522333 10.1186/1471-2288-13-48 PMC3614526

[23] Arksey H O’Malley L Scoping studies: towards a methodological framework. *Int J Soc Res Methodol* 2005; 8:19–32.

[24] Levac D Colquhoun H O’Brien KK Scoping studies: advancing the methodology. *Implement Sci* 2010; 5:69. 20854677 10.1186/1748-5908-5-69 PMC2954944

[25] Miyashita M Morita T Shima Y Nurse views of the adequacy of decision making and nurse distress regarding artificial hydration for terminally ill cancer patients: a nationwide survey. *Am J Hosp Palliat Care* 2008; 24:463–469. 10.1177/1049909107302301 17601838

[26] Murakawa Y Nihei Y Understanding the concept of a “good death” in Japan: differences in the views of doctors, palliative and non-palliative ward nurses. *Int J Palliat Nurs* 2009; 15:282–289. 19568215 10.12968/ijpn.2009.15.6.42987

[27] Nakazawa K Kizawa Y Maeno T Palliative care physicians’ practices and attitudes regarding advance care planning in palliative care units in japan: a nationwide survey. *Am J Hosp Palliat Care* 2013; 31:699–709. 24113194 10.1177/1049909113507328

[28] Shimoinaba K Lee S Support for nurses working with palliative care patients in Japan. *Asian J Nurs* 2007; 10:243–250.

[29] Shimoinaba K O’Connor M Lee S Losses experienced by Japanese Nurses and the way they grieve. *J Hosp Palliat Nurs* 2014; 16:224–230.

[30] Santisteban Etxeburu I Mier O A descriptive study of death anxiety and stressors in the various practitioners of a Palliative Care Unit. *Med Paliativa* 2006; 13:18–24.

[31] Ablett JR Jones RSP Resilience and well-being in palliative care staff: a qualitative study of hospice nurses’ experience of work. *Psychooncology* 2007; 16:733–740. 17171723 10.1002/pon.1130

[32] Zinn CL Moriarty D Nurses’ perceptions of palliative sedation in a scottish hospice: An exploratory study. *J Hosp Palliat Nurs* 2012; 14:358–364.

[33] Evans MJ Hallett CE Living with dying: a hermeneutic phenomenological study of the work of hospice nurses. *J Clin Nurs* 2007; 16:742–751. 17402956 10.1111/j.1365-2702.2006.01620.x

[34] Martins C Basto ML Relieving the suffering of end-of-life patients. *J Hosp Palliat Nurs* 2011; 13:161–171.

[35] Tornøe KA Danbolt LJ Kvigne K The power of consoling presence—hospice nurses’ lived experience with spiritual and existential care for the dying. *BMC Nurs* 2014; 13:25. 25214816 10.1186/1472-6955-13-25 PMC4160718

[36] Gama G Barbosa F Vieira M Meaning of life in nurses caring for patients at the end of life. *Eur J Palliat Care* 2014; 21:130–135.

[37] Källström Karlsson IL Ehnfors M Ternestedt B-M Five nurses’ experiences of hospice care in a long-term perspective. *J Hosp Palliat Nurs* 2008; 10:224–232.

[38] Oliveira AC Sa L Silva MJP The attitude of nurses face the autonomy of the terminal ill patient. *Rev Bras Enferm* 2007; 60:286–290. 17684905 10.1590/s0034-71672007000300007

[39] Oliveira AC Silva MJP Autonomy in palliative care: concepts and perceptions of a health teamwork. *Acta Paul Enferm* 2010; 23:212–217.

[40] Gama G Barbosa F Vieira M Personal determinants of nurses’ burnout in end of life care. *Eur J Oncol Nurs* 2014; 18:527–533. 24888265 10.1016/j.ejon.2014.04.005

[41] Gama G Barbosa F Vieira M Factors influencing nurses’ attitudes toward death. *Int J Palliat Nurs* 2012; 18:267–273. 22885899 10.12968/ijpn.2012.18.6.267

[42] Martens ML A comparison of stress factors in home and inpatient hospice nurses. *J Hosp Palliat Nurs* 2009; 11:144–153.

[43] Henoch I Browall M Melin-Johansson C The Swedish version of the Frommelt Attitude Toward Care of the Dying scale: aspects of validity and factors influencing nurses’ and nursing students’ attitudes. *Cancer Nurs* 2014; 37:E1–E11. 10.1097/NCC.0b013e318279106b 23357885

[44] Bezerra do Amaral J Menezes Mdo Rde Martorell-Poveda Antonia M Ethic and bioethic dilemmas on palliative care for hospitalized elderly: nurses’ experience. *Cult los Cuid* 2012; 16:14–21.

[45] Dee JF Endacott R Doing the right thing at the right time. *J Nurs Manag* 2011; 19:186–192. 21375621 10.1111/j.1365-2834.2010.01200.x

[46] Timmermann M Naziri D Etienne A-M Defence mechanisms and coping strategies among caregivers in palliative care units. *J Palliat Care* 2009; 25:181–190. 19824279

[47] Thornburg P Schim SM Paige V Nurses’ experiences of caring while letting go. *J Hosp Palliat Nurs* 2008; 10: 382–391.

[48] Scott C Nurses worried about the erosion of the caring role. Scott H, ed. *Br J Nurs* 2004; 13:348–1348. 15150470 10.12968/bjon.2004.13.7.12654

[49] Pereira SM Cuidados Paliativos. Confrontar a Morte. Lisboa:Universidade Católica Editora; 2010.

[50] Capelas M Silva S Alvarenga M Desenvolvimento histórico dos Cuidados Paliativos: visão nacional e internacional. *Cuid Paliativos* 2014; 1:7–13.

[51] Worldwide Palliative Care Alliance Global Atlas of Palliative Care at the End of Life. London:Worldwide Palliative Care Alliance; 2014.

[52] Mays N Roberts E Popay J Fulop N Allen P Clarke A Black N Synthesising research evidence. *Studying the Organisation and Delivery of Health Services: Research Methods*. London:Routledge; 2001 188–220.

[53] Parola V Coelho A Cardoso D Burnout in palliative care settings compared to other settings: a systematic review. *J Hosp Palliat Nurs* 2017; 19 (5.):

